# Intestinal Resident Yeast *Candida glabrata* Requires Cyb2p-Mediated Lactate Assimilation to Adapt in Mouse Intestine

**DOI:** 10.1371/journal.pone.0024759

**Published:** 2011-09-09

**Authors:** Keigo Ueno, Yasuhiko Matsumoto, Jun Uno, Kaname Sasamoto, Kazuhisa Sekimizu, Yuki Kinjo, Hiroji Chibana

**Affiliations:** 1 Medical Mycology Research Center (MMRC), Chiba University, Chiba, Japan; 2 Laboratory of Microbiology, Graduate School of Pharmaceutical Sciences, The University of Tokyo, Tokyo, Japan; 3 Laboratory of Immune Regulation, Department of Chemotherapy and Mycoses, National Institute of Infectious Diseases, Tokyo, Japan; University of Missouri-Kansas City, United States of America

## Abstract

The intestinal resident *Candida glabrata* opportunistically infects humans. However few genetic factors for adaptation in the intestine are identified in this fungus. Here we describe the *C. glabrata CYB2* gene encoding lactate dehydrogenase as an adaptation factor for survival in the intestine. *CYB2* was identified as a virulence factor by a silkworm infection study. To determine the function of *CYB2*, we analysed *in vitro* phenotypes of the mutant Δ*cyb2*. The Δ*cyb2* mutant grew well in glucose medium under aerobic and anaerobic conditions, was not supersensitive to nitric oxide which has fungicidal-effect in phagocytes, and had normal levels of general virulence factors protease, lipase and adherence activities. A previous report suggested that Cyb2p is responsible for lactate assimilation. Additionally, it was speculated that lactate assimilation was required for *Candida* virulence because *Candida* must synthesize glucose via gluconeogenesis under glucose-limited conditions such as in the host. Indeed, the Δ*cyb2* mutant could not grow on lactate medium in which lactate is the sole carbon source in the absence of glucose, indicating that Cyb2p plays a role in lactate assimilation. We hypothesized that Cyb2p-mediated lactate assimilation is necessary for proliferation in the intestinal tract, as the intestine is rich in lactate produced by bacteria flora, but not glucose. The Δ*cyb2* mutant showed 100-fold decreased adaptation and few cells of *Saccharomyces cerevisiae* can adapt in mouse ceca. Interestingly, *C. glabrata* could assimilate lactate under hypoxic conditions, dependent on *CYB2*, but not yeast *S. cerevisiae*. Because accessible oxygen is limited in the intestine, the ability for lactate assimilation in hypoxic conditions may provide an advantage for a pathogenic yeast. From those results, we conclude that Cyb2p-mediated lactate assimilation is an intestinal adaptation factor of *C. glabrata*.

## Introduction

*Candida albicans* and *Candida glabrata* are commensal yeasts that live in association with human mucosal surfaces including the intestine and vagina, and often turn pathogenic in immnunocompromised individuals, such as AIDS, leukemia or diabetes patients and cause a high rate of mortality [1], [2], [3], [4]. The number of effective antifungal drugs is limited, and resistance to frequently-used drugs including echinocandins is emerging [5]. Novel drug targets are needed.

Armed with the complete genome sequence and using reverse genetics, it has been revealed that *Candida* pathogens have many factors required for infection. Those factors that are not conserved in humans are suggested as potential antifungal drug targets. Isocitrate lyase (Icl1p) is a microbe specific enzyme and involved in the glyoxylate cycle and gluconeogenesis (Figure S1; simple pathway map or see review [6], [7]). The *C. albicans* Δ*icl1* mutant is attenuated for virulence in a mouse model of systemic candidiasis [8]. This report concludes that the glyoxylate cycle is a critical adaptation factor for *C. albicans* to survive in the host environment, especially in the macrophage where nutrient availability is low for pathogens. The glyoxylate cycle enables acetyl-CoA to be used as a source of carbon. Hence it was proposed that fatty acids β-oxidation producing acetyl-CoA is necessary for the glyoxylate cycle in *C. albicans* (Figure S1). Indeed, genes acting in β-oxidation and the glyoxylate cycle are upregulated during macrophage phagocytosis [9]. Unexpectedly and unlike the glyoxylate cycle, peroxisomal fatty acid β-oxidation was not essential for virulence [7]. This report speculated that the lactate assimilation as an alternative pathway can support the glyoxylate cycle, because the transcripts for *CYB2* (encoding L-lactate dehydrogenase, converting L-lactate to pyruvate), *JEN1* and *JEN2* (lactate transporters and its homolog) were induced 15.7-fold, 5.5-fold and 159.5-fold, respectively, in macrophages [10] (Figure S1). To clarify this speculation, the Δ*jen1/*Δ*jen2* double knock out mutant of *C. albicans* was constructed and analysed for virulence. Unexpectedly, the double mutant was not attenuated for virulence in the mouse [11]. Thus, the importance of lactate assimilation in disease is unclear.

The model of disseminated Candidiasis has been mainly used to study virulence factor in *Candida* species. Despite *Candida* pathogens being intestinal residents, only a few intestinal infection studies have been reported [12]. Consequently, the intestinal adaptation factors of *Candida* remain to be clarified. For protection of *Candida* infection in immunocompromised patients, it is important to control the fungal burden within the intestine. Thus it is of significance to elucidate the intestinal adaptation factors of *Candida*, potentially leading to proposal of novel antifungal drug targets.

In this context, we screened virulence-attenuated mutants from our recombinant collection using silkworm infection to identify a novel virulence factor. In the process of this screening we found a *cyb2* disruptant. The Δ*cyb2* strain could not grow on medium containing L-lactate as a sole carbon source, and showed 100 fold decreased adaptation in mouse cecum. Interestingly, *C. glabrata* could assimilate lactate under hypoxic conditions, dependent on *CYB2*. Because accessible oxygen is limited in the intestine, the ability of lactate assimilation in hypoxic conditions may provide an advantage for a pathogenic yeast. We conclude that Cyb2p-mediated lactate assimilation is an intestinal adaptation factor of *C. glabrata*.

## Results

### Identification of a virulence-attenuated cyb2 mutant with a silkworm infection model

In the process of screening for virulence-attenuated mutants from our systemic recombinant collection with a hyperglycemic silkworm infection model [13], [14], [15], [16], we found a *cyb2* mutant (KUE11538) that was attenuated for virulence. On an average, the LD_50_ of the *cyb2* mutant was 2×10^8^ CFU/larvae, a 4.5 fold increase compared to the control strain (KUE100_chr464).

**Figure 1:**
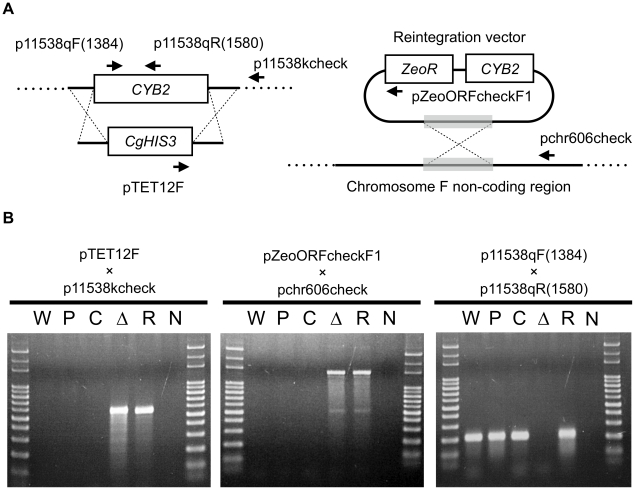
Construction of the CYB2 deletion and reintegration strains. (A) Deletion and reintegration. CYB2 ORF was replaced by CgHIS3 marker, and CYB2 reintegration was performed with pZeoi_CgCyb2 into the non-coding region on chromosome F. The arrows represent three primer sets used to confirm strains. (B) Confirmation of deletion and reintegration. Three PCRs were done for the confirmation. W: Wild type strain CBS138, P: Parent strain KUE100, C: Control strain KUE100_chr606, Δ: KUE11538BV deletant strain, R: KUE11538CV revertant strain, N: Non-template control.

The gene CAGL0K10736g encodes a peptide with 64.97% amino acids sequence identity to the L-lactate dehydrogenase (LDH, Cyb2p) in *S. cerevisiae*. We will thus refer to this gene as *CYB2* hereafter (Figure S2). Cyb2p includes three domains, a transit signal domain for the targeting to mitochondria (aa 1 - 79), a heme binding domain (aa 88 - 165), and a flavin mononucleotide (FMN) hydroxy acid dehydrogenase domain (aa 197 - 563). The transit signal domain had low homology but the two other domains were highly conserved (Figure S2). To confirm the function of *CYB2* in *C. glabrata* and for use in subsequent experiments, we constructed a null mutant (KUE11538BV) by replacement of the entire ORF by *CgHIS3* gene and a revertant (KUE11538CV) by reintegration of *CYB2* ORF with 500-bp promoter and 200-bp terminator with a zeocin marker (Figure 1).

**Figure 2:**
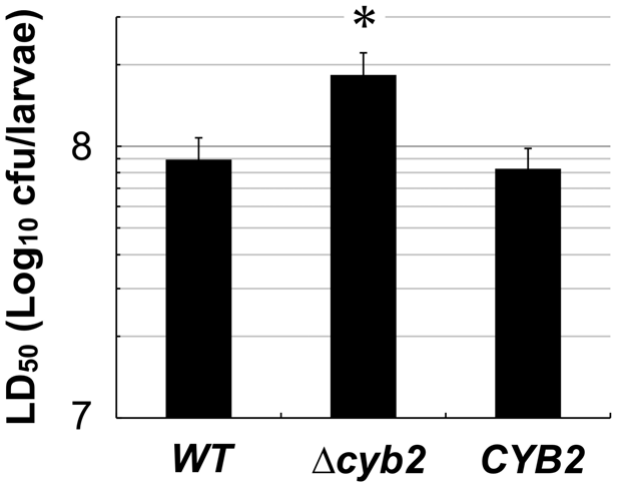
The virulence of Δcyb2 strain is attenuated in a silkworm infection model. Ten hyperglycemic larvae were used, and all infected larvae were kept in 37°C. LD_50_ was determined at 24 h after injection. Three experiments were independently carried out. Asterisks: *p*<0.05 with Student t-test vs. *WT* or *CYB2*. *WT*; CBS138 wild type strain, Δ*cyb2*: KUE11538BV deletant strain, *CYB2*: KUE11538CV revertant strain.

To confirm the virulence attenuation caused only by the gene deletion, a *CYB2* revertant was included in the infection study. The virulence of the Δ*cyb2* strain was significantly attenuated compared to to the wild type strain (CBS138) and the revertant strains (Figure 2).

### CYB2 is not required for the growth in glucose conditions

**Figure 3:**
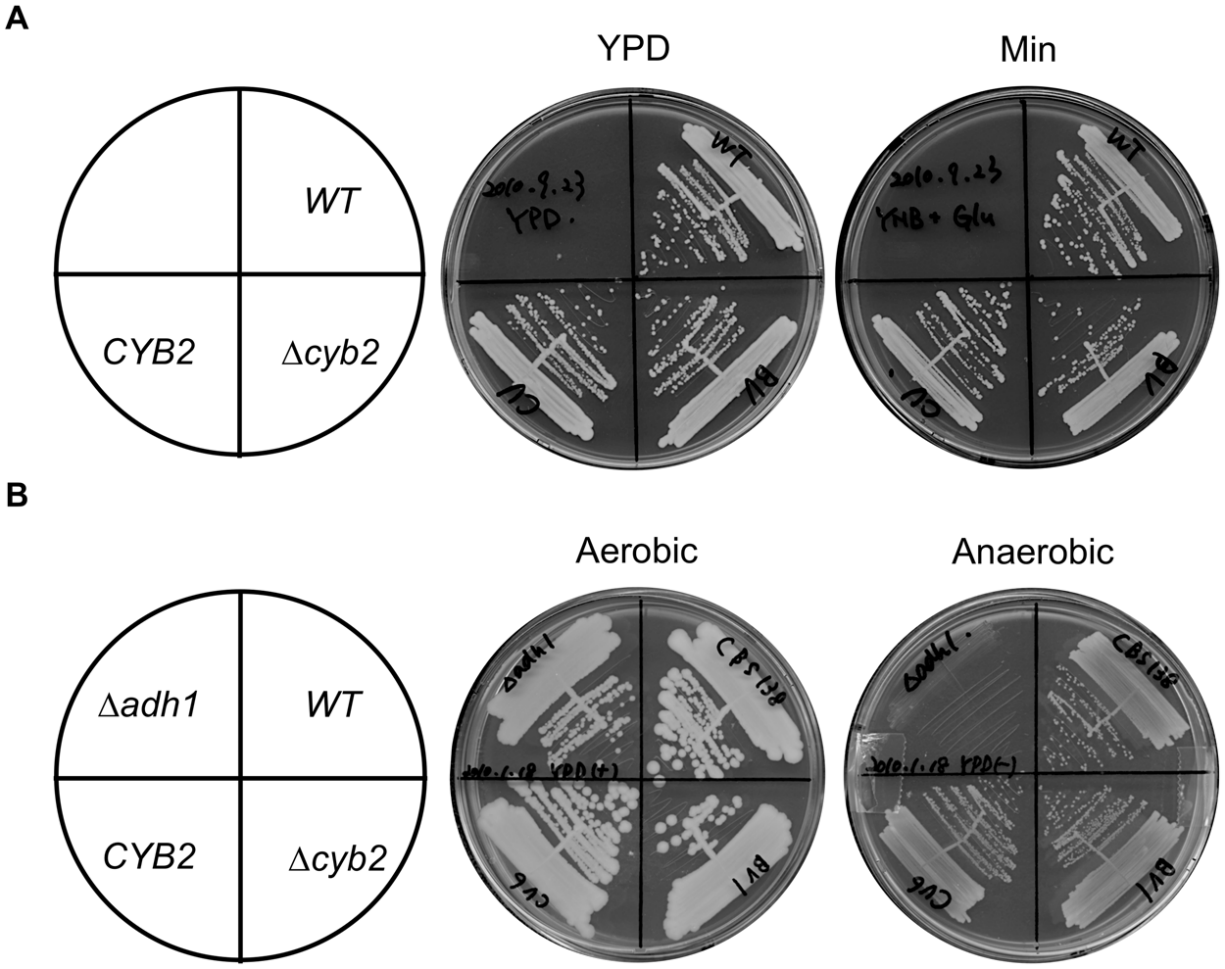
CYB2 is not essential for growth in glucose condition. (A) The Δcyb2 strain showed normal aerobic growth on glucose medium. YPD and Min medium show growth after 1 day and 2 days at 37°C, respectively. (B) ADH1 was essential for growth in anaerobic condition. The plates show growth after 2 days at 37°C. *WT*: CBS138 wild type strain, Δ*cyb2*: KUE11538BV deletant strain, *CYB2*: KUE11538CV revertant strain, Δ*adh1*: KUE13139 deletant strain.

Slow growth of the Δ*cyb2* strain may lead to virulence attenuation. To test this possibility, we evaluated the growth of the Δ*cyb2* strain in nutrient rich media (YPD) or minimal media (Min) in aerobic conditions. The Δ*cyb2* strain grew equally well as the wild type strain and the revertant strain (Figure 3A). Doubling time of Δ*cyb2* strain in YPD liquid medium was 51.3±0.2 min, with no significant difference to wild type strain (48.7±1.7 min) or the revertant strain (50.9±0.6 min).

The ability to grow under anaerobic condition also is important for infection [17]. In the Δ*cyb2* strain, a defect in growth under hypoxic conditions may lead to virulence attenuation, because Cyb2p may play a role in lactate fermentation which is important for anaerobic growth as well as alcohol fermentation. To test the Cyb2p-dependent anaerobic growth, Δ*cyb2* and Δ*adh1* strain were cultivated on YPD under anaerobic condition. *ADH1* (CAGL0I07843g) is an orthologue of *S. cerevisiae ADH1* encoding alcohol dehydrogenase which is responsible for alcohol fermentation [18]. The Δ*cyb2* strain, but not Δ*adh1* strain, could grow on YPD plate under anaerobic conditions (Figure 3B). These results showed that the *CYB2* is not essential for aerobic or anaerobic growth, while the *ADH1* is essential for anaerobic growth. Additionally, the virulence attenuation is not accounted for by a growth defect due to low oxygen availability.

### The Δcyb2 strain shows wild type sensitivity to nitric oxide

Nitric oxide is known to kill pathogens in the phagocyte [19]. For example, the lactate dehydrogenases encoded by *ldh1* and *ldh2* of *Staphylococcus aureus* are required for tolerance towards nitric oxide, and the Δ*ldh1*Δ*ldh2* double mutant could not survive in macrophages and showed reduced virulence [20]. We hypothesized that the virulence attenuation of Δ*cyb2* strain was due to increased sensitivity towards nitric oxide. To test this, we exposed Δ*cyb2* mutant to DETA NONOate which generates nitric oxide. No supersensitivity of Δ*cyb2* strain was found, Minimum inhibitory concentration (MIC) for DETA NONOate were 0.95 mg/ml for the Δ*cyb2* strain and wild type strain. This indicates that lactate dehydrogenase *CYB2* is not essential for tolerance towards nitric oxide unlike *S. aureus*, and that could not explain virulence attenuation of Δ*cyb2* strain.

### The Δcyb2 strain has normal activities of protease, lipase and adherence

**Figure 4:**
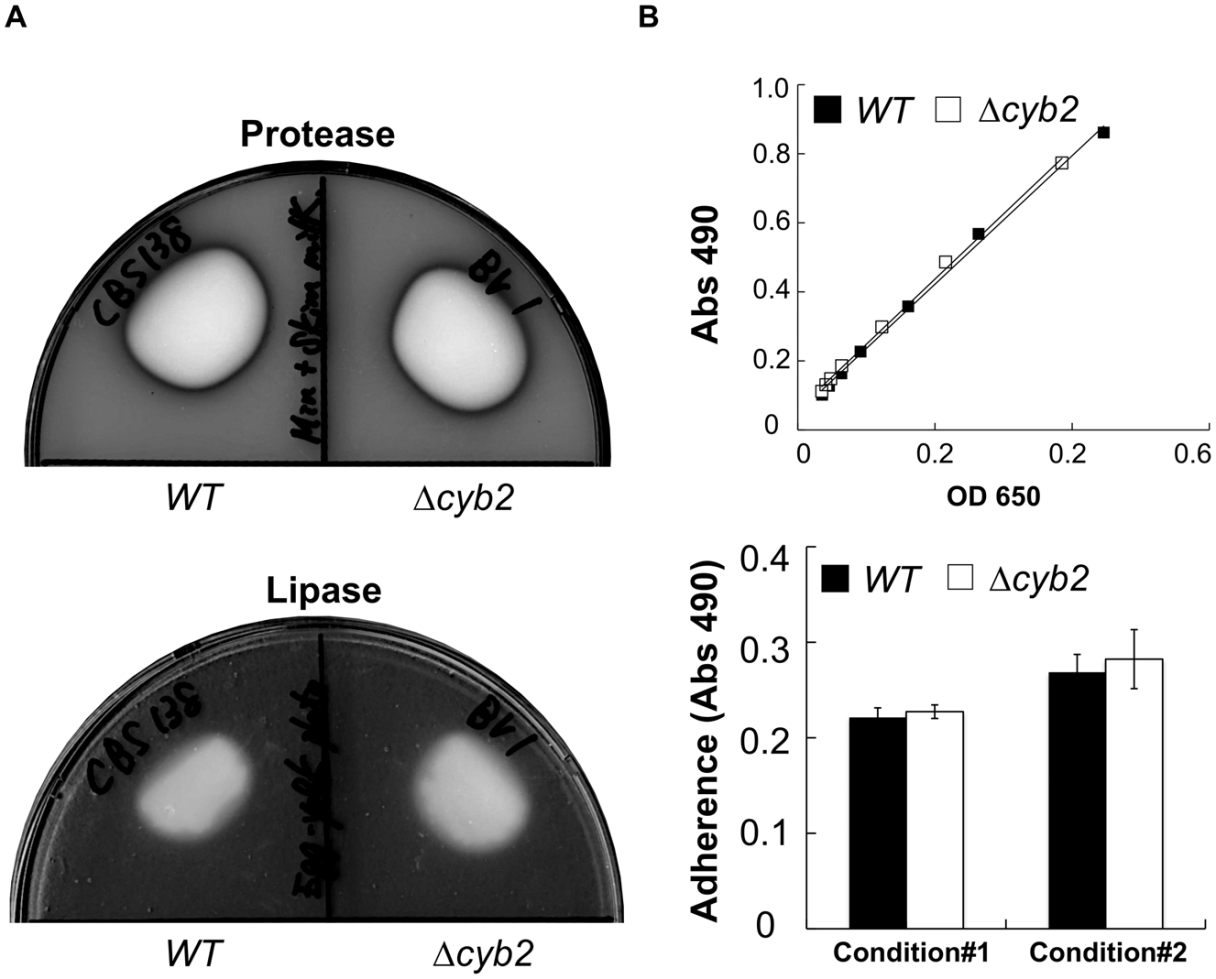
The Δcyb2 strain do not have defects in general virulence traits. (A) Normal activities of protease and lipase in Δcyb2 strain. The halos surrounding the colonies represent clearing of the casein by secreted protease and precipitation of calcium complex with fatty acid after lipase digestion of lipid in the egg yolk. The photographs show the results after 2 days at 37°C. (B) Comparable adherence in wild type and Δcyb2 strain. The graph (under panel) shows adherence of cells to polystyrene measured as the absorbance of XTT at 490nm. Overnight culture grown in the *CYB2-*induction medium (OD_600_  =  10 or 20 indicates condition #1 and #2) was applied to the polystyrene wells at the concentrations indicated. A control experiment (upper panel) was performed to confirm an equal metabolization of XTT between *WT* and Δ*cyb2* strain. OD_650_ and A_490_ showed cell numbers and metabolization level of XTT, respectively. *WT*: CBS138 wild type strain, Δ*cyb2*: KUE11538BV deletant strain.

The attenuated virulence of Δ*cyb2* stain may be due to indirect reductions in other virulence such as protease or lipase activity, or adherence. However the Δ*cyb2* strain showed comparable activity for protease, lipase and adherence comparable to the wild type strain (Figure 4). These findings also can not explain virulence attenuation of Δ*cyb2* strain.

### CYB2 is required for lactate assimilation under glucose starvation

**Figure 5:**
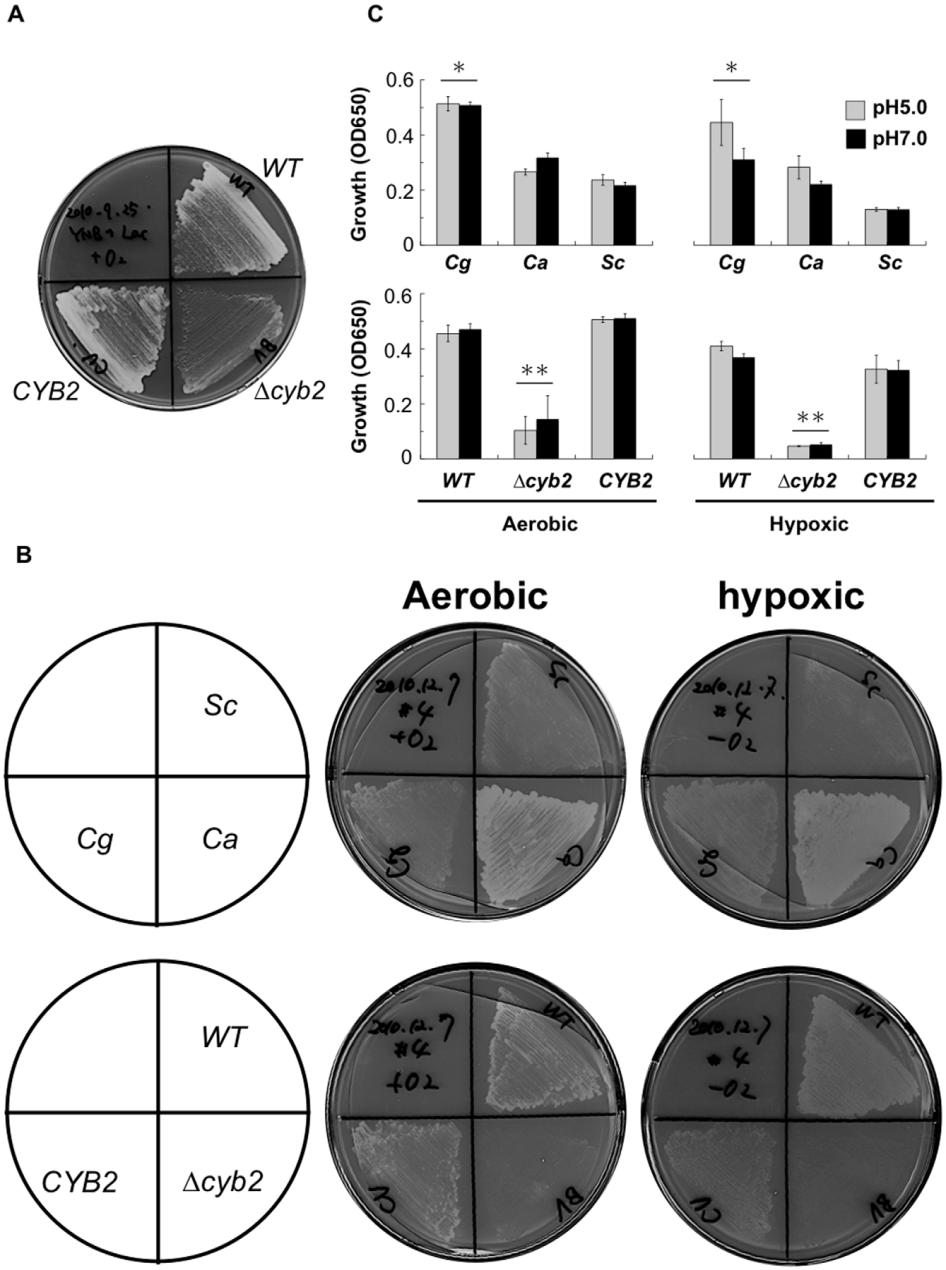
CYB2 plays a role in lactate assimilation under glucose starvation. (A) Growth defect of Δ**cyb2 strain in lactate as the sole carbon source. The lactate plate (2% agar, pH 5.0; optimized empirically for the aerobic plate assay) was aerobically cultivated for 3 days at 37°C. (B) C. glabrata and C. albicans could grow on the lactate plate under hypoxic condition. For hypoxic cultivations, the lactate plates (4% agar, pH 7.0; optimized empirically for the hypoxic plate assay) were cultivated in absolutely anaerobic condition for 6 days at 37°C, and then were quickly moved into microaerophilic condition for 3 days at 37°C. (C) Lactate assimilation in the liquid medium. Monocarboxylic acids are partially dissociated in aqueous solution, according to their pKa(s) and to the medium pH. The anionic form of these compounds is important for uptake by transporters such as Jen1p [45], [46]. The medium containing 1% L-lactate and 2% L-lactate were used to compare three species *C. glabrata*, *C. albicans* and *S. cerevisiae* (upper panel), and for three strains, wild type, deletant and revertant strains (lower panel), respectively. The 2% lactate medium was not suite for growth of *S. cerevisiae*. The 96-well plates were incubated for 12 days at 37°C. This is representative data from three independent experiments. Asterisk; *p*<0.04 with Student t-test vs. *Ca* or *Sc,* Double asterisk*; p*<0.003 with Student t-test vs. *WT* or *CYB2, Sc*: *S. cerevisiae* standard strain S288C, *Cg*: C. *glabrata* standard strain CBS138, *Ca*: *C. albicans* standard strain SC5314, *WT*; CBS138 wild type strain, Δ*cyb2*: KUE11538BV deletant strain, *CYB2*: KUE11538CV revertant strain.

A previous study speculated that Cyb2-dependent lactate assimilation plays a role in the virulence of *C. albicans,* because lactate assimilation can support the glyoxylate cycle and gluconeogenesis under glucose-limited conditions such as in the host [7]. To validate this hypothesis, we tested growth of the Δ*cyb2* strain in a lactate medium with no other carbon sources. This forces the cell to make glucose through gluconeogenesis from lactate. The wild type strain and the revertant strain grew well on lactate medium, but the Δ*cyb2* strain did not (Figure 5A). In addition to being a carbon source, high concentrations of lactate are toxic to cells. MICs of L-lactate against these strains were equal (50 mM). This indicates that Cyb2p played a role in lactate assimilation, but not in lactate detoxification.

Oxygen availability is limited for pathogens within the host [21], [22]. Since the virulence of the Δ*cyb2* mutant was attenuated in silkworm, we hypothesize that *C. glabrata* Cyb2p can work in hypoxic conditions as well as aerobic condition. To test this possibility, we observed growth of three yeasts *S. cerevisiae*, *C. glabrata* and *C. albicans* on lactate as the sole carbon source under hypoxic conditions. *S. cerevisiae* does not express lactate dehydrogenase (Cyb2p) in anaerobic conditions [23]. All three yeasts grew in aerobic conditions, while *C. glabrata* and *C. albicans* grew in oxygen limiting conditions (Figure 5B). Similarly, *C. glabrata* and *C. albicans* grew better than *S. cerevisiae* in the lactate liquid medium under hypoxic condition (Figure 5C). This result shows that these yeasts can assimilate L-lactate under hypoxic conditions and can retain expression/function of Cyb2p. Furthermore these findings agree with previous speculation [7] and suggest that the defect of lactate assimilation can cause virulence attenuation.

### The Δcyb2 strain is less adapted in the mouse cecum

**Figure 6:**
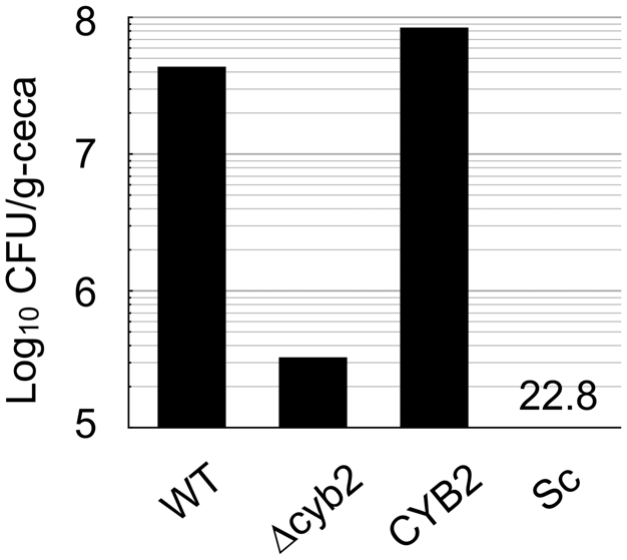
The Δcyb2 strain is less adapted to mouse cecum. Each strain was intragastrically inoculated by using a metallic gastric tube. Then 13 days after the inoculation, three hyperglycemic mice having average body weight in the group were euthanized. The three ceca were extirpated without washing inner feces, homogenized together, and spread onto the YRM plates to determine the number of CFU of each strain in ceca (wet weight). This is representative data from two independent experiments. Abbreviation and infection dose are as follows; *WT: C. glabrata* CBS138 (5.3×10^7^ cfu/mouse), Δ*cyb2:* KUE11538BV deletant strain (2.5×10^7^ cfu/mouse), *CYB2:* KUE11538CV revertant strain (3.8×10^7^ cfu/mouse) and *Sc: S. cerevisiae* S288C (5.7×10^7^ cfu/mouse).

Lactate is produced by intestinal bacterial flora and is an abundant carbon source in the intestine [24], [25]. On the other hand, glucose is a preferred carbon source, but the availability of glucose is low because of strong competition for this carbon source amongst the intestinal cells, bacterial flora and *Candida*. We predicted that Cyb2p-dependent lactate assimilation is required for adaptation in the intestinal tract. We performed an intestinal infection study using C57BL/6JJcl mice. According to a previous report, *C. albicans* highly accumulates in cecum and the fungal burden in the cecum reflects intestinal fungal colonization [26], and *C. glabrata* can colonize in the cecum [27]. Thus we measured fungal burden in the cecum. Four strains, *C. glabrata* wild type strain, Δ*cyb2* deletant strain, the revertant strain and a *S. cerevisiae* wild type strain (S288C), were intragastrically inoculated and the accumulation of these strains was measured in ceca 13 days after infection. We found that CFU of Δ*cyb2* strain from ceca was more than 100 fold decreased compared to the wild type and revertant strains (Figure 6). Few *S. cerevisiae* colony forming units (CFU) could be isolated from the ceca (Figure 6). This result shows that Cyb2p is required for adaptation in an intestine, and suggests that Cyb2p-dependent lactate assimilation is required to adapt in this glucose-limited environment of the host.

### C. glabrata prefers L-lactate as an alternative carbon source

**Figure 7:**
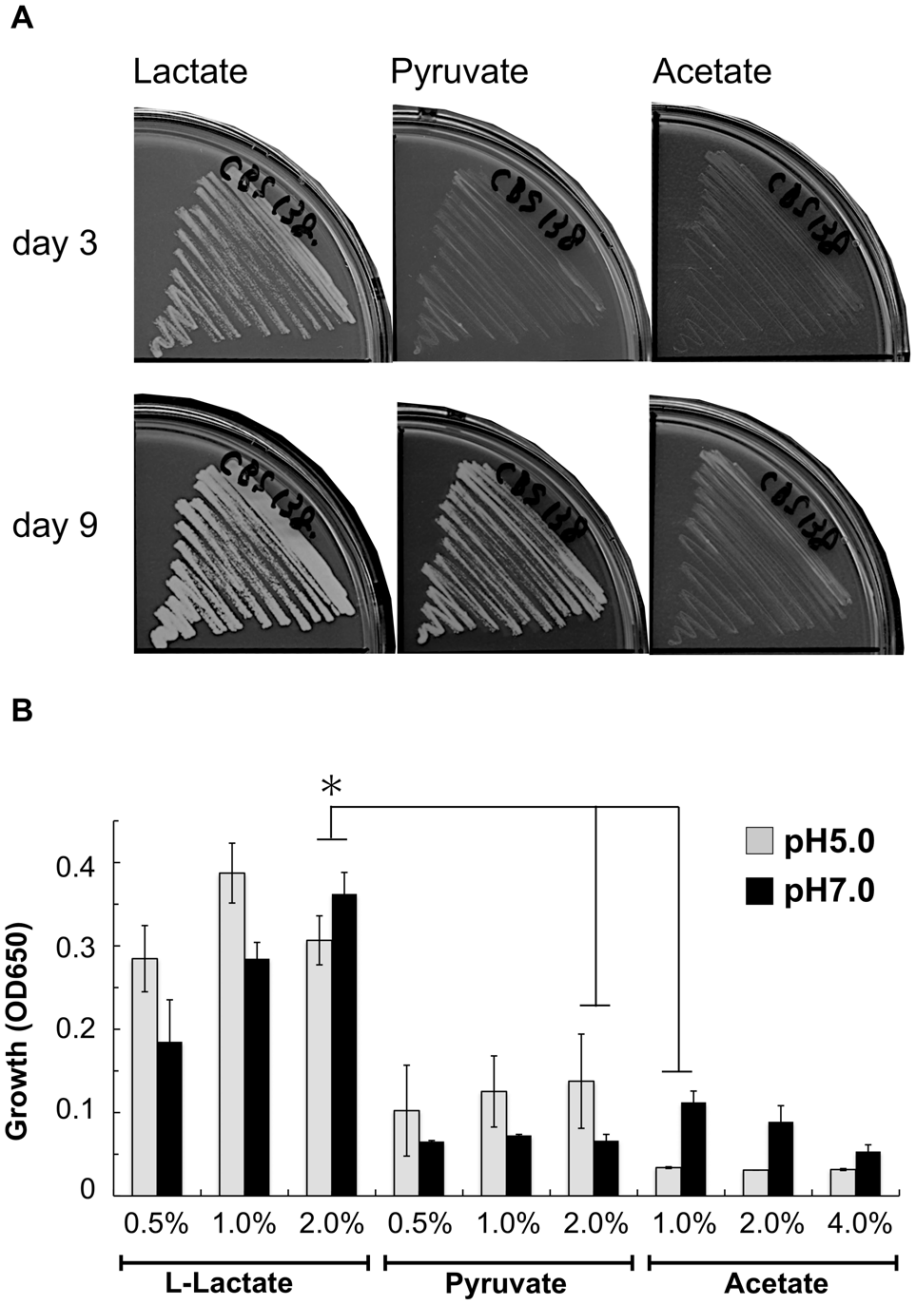
C. glabrata prefers L-lactate as an alternative carbon source. (A) Agar plate assay. The lactate, pyruvate and acetate plate pH 7.0 contains 2% L-lactate, 4% sodium acetate trihydrate and 2% pyruvate as a sole carbone source, respectively. C. glabrata CBS138 was cultivated at 37°C for 9 days. (B) Liquid medium assay. The 96-well plates were incubated for 6 days at 37°C. This is representative data from three independent experiments. Asterisk; *p*<0.05 with Student t-test.

Other organic acids besides L-lactate also exist in the intestine, for example an acetate and pyruvate that are produced by intestinal bacteria [25]. These organic acids can serve as alternative carbon sources to fuel gluconeogesesis via the glyoxylate cycle [6], [28]. Because *C. glabrata* still could adapt in the cecum without lactate assimilation (Figure 6), *C. glabrata* might assimilate other organic acids in the cecum. In contrast, the fact of *CYB2*-dependent adaptation suggested that *C. glabrata* prefers lactate as an alternative carbon source. To test this possibility, we compared *C. glabrata* growth in lactate, pyruvate and acetate medium. *C. glabrata* grew better in the lactate medium than the pyruvate or acetate medium (Figure 7). With 9 days incubation, *C. glabrata* could grow on the pyruvate and acetate plate (Figure 7A). This result suggests that *C. glabrata* can assimilate pyruvate and acetate as well as lactate, but lactate is required for initial proliferation of *C. glabrata* under glucose-limited conditions.

### Hap2p and Hap5p transcription factors are required for lactate assimilation and virulence

**Figure 8:**
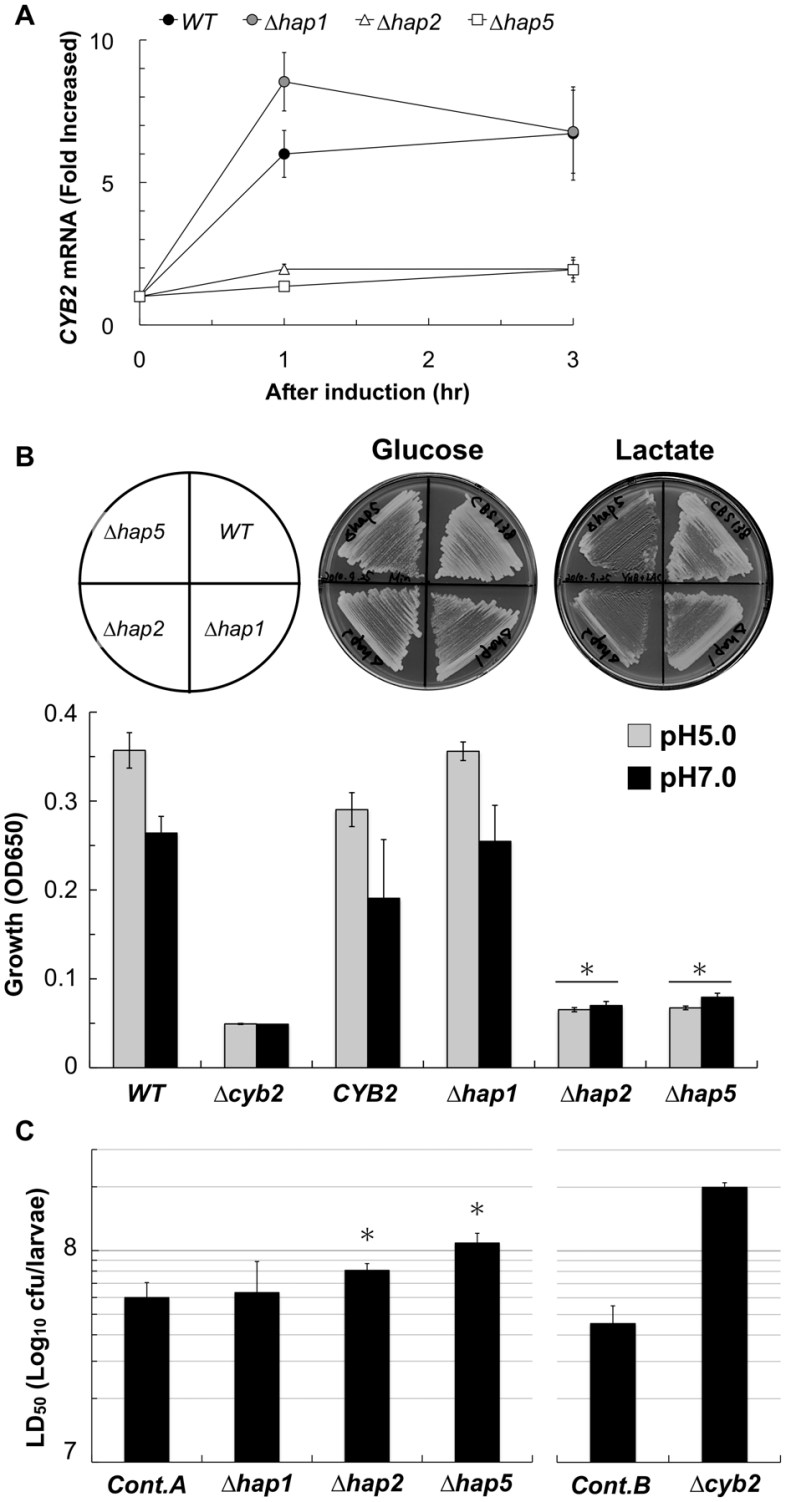
Hap2p and Hap5p transcription factors are required for lactate assimilation and virulence. (A) CYB2 transcription was not fully activated in Δhap2 and Δhap5 strains. The *CYB2*-induction medium [*29*] was used to activate *CYB2* transcription. Transcription was evaluated by qRT-PCR. This is representative data from three independent experiments. (B) Lactate assimilation was decreased in Δhap2 and Δhap5 strains. Strains were cultivated on lactate plates (2% agar,pH 5.0) for 3 days at 37°C (Upper panel), and in liquid medium containing 2% L-lactate for 5 days at 37°C. This is representative data from three independent experiments. Asterisk; *p*<0.002 with Student t-test vs. *WT, WT*: CBS138 wild type strain, Δ*cyb2*: KUE11538BV deletant strain, *CYB2*: KUE11538CV revertant strain, Δhap1: KUE16015, Δhap2: KUE13633, Δ*hap5*: KUE14440. (C) The virulence of Δhap2 or Δhap5 strains was attenuated in a silkworm infection model. Ten hyperglycemic larvae were used, and all infected larvae were kept in 37°C. LD_50_ was determined at 24 h after injection. Three experiments were independently carried out. Asterisks: *p*<0.05 with Student t-test vs. *Cont.A*. *Cont.A*: KUE100_chr606 control strain, Δ*hap1*: KUE16015, Δ*hap2*: KUE13633, Δ*hap5*: KUE14440, *Cont.B*; KUE100_chr464 control strain, Δ*hap2*; KUE11538 deletant strain.

In *S. cerevisiae*, transcription of *CYB2* is activated by *trans*-acting activator Hap1p and *cis*-acting activator complex Hap2/3/4/5 sensing glucose starvation [29]. Bioinformatic prediction: MATCH™ (http://www.gene-regulation.com/) revealed a potential binding site of the Hap2/3/4/5p complex in the -177 ∼ -162 region of the *CgCYB2* promoter, including the CCAAT recognition sequence of Hap2/3/4/5p. To analyse the functions of Hap transcriptional factors in lactate assimilation, we constructed *C. glabrata* deletion mutants Δ*hap1* (CAGL0K05841g), Δ*hap2* (CAGL0H07843g), Δ*hap5* (CAGL0K09900g). Quantitative RT-PCR (qRT-PCR) analysis revealed that Δ*hap2* and Δ*hap5* strains could not fully activate *CYB2* transcription in glucose-limited condition (Figure 8A). Although all deletants grew as well as the wild type strain in glucose media, Δ*hap2* and Δ*hap5* strains grew worse than wild type strain in the lactate medium, but not Δ*hap1* strain (Figure 8B). Furthermore the virulence of Δ*hap2* and Δ*hap5* strains were significantly attenuated in a silkworm infection model (Figure 8C). These findings suggest that Hap2p and Hap5p transcription factors are required for lactate assimilation and virulence.

## Discussion

This study demonstrates that Cyb2p plays an essential role in lactate assimilation and identifies it as a novel adaptation factor for survival in the intestine, as well as substantiating the previous speculation that lactate assimilation is required for *Candida* infection [7].

The ability to assimilate lactate under glucose-limited conditions is a feasible explanation for the result in which the Δ*cyb2* strain is less adapted to the cecum (Figure 6). The Δ*cyb2* strain grew well in glucose medium under aerobic and anaerobic condition, was not supersensitive to nitric oxide and had normal levels of general virulence factors like protease, lipase, adherence activities (Figure 3, 4). These findings could not explain the reduced fitness of the Δ*cyb2* strain in the cecum. Instead, the Δ*cyb2* strain could not assimilate L-lactate under aerobic and hypoxic conditions (Figure 5). Accessible glucose is limited in the cecum due to absorption by the host [30], [31], but lactate is supplied by intestinal bacteria and its concentration is higher than other organic acids [25]. Furthermore *C. glabrata* grew more rapidly in lactate medium than acetate and pyruvate medium, indicated that *C. glabrata* prefers lactate for initial proliferation (Figure 7). Interestingly, *C. albicans* and *C. glabrata* grew better than *S. cerevisiae* in lactate medium under hypoxic conditions (Figure 5B and 5C). Because *Candida* should face hypoxic stress in the host, the ability for lactate assimilation under hypoxic conditions might provide an advantage during infection. However this trait can not fully account for few *S. cerevisiae* colonization in the cecum (Figure 6). It seems that *S. cerevisiae* has crucially weak virulence trait(s) such as stress response and adherence to epithelial cells. Lactate is not only a nutrient source but also an anti-microbial molecule associated with the mucosa [24], [25]. Another possibility is that Cyb2p plays a role in the detoxication of lactate via lactate consumption. However the Δ*cyb2* strains was not supersensitive to lactate, indicating little or no contribution of Cyb2p to lactate detoxication. Thus we conclude that lactate assimilation is required for *C. glabrata* to survive in the intestine, and Cyb2p is an intestinal adaptation factor.

To identify regulatory mechanisms of lactate assimilation, it is meaningful to understand the nutrient acquisition system in the process of infection. In macrophages, transcription of *C. albicans CYB2* was 15.7-fold upregulated [10]. Transcription of *S. cerevisiae CYB2* is activated by the Hap family transcriptional activator, Hap1p and Hap2/3/4/5p complex, responding to a glucose starvation signal [29]. In *C. glabrata*, Δ*hap2* or Δ*hap5* strains could not fully activate *CYB2* transcription under glucose-limited condition and grew worse than the wild type strain in lactate medium (Figure 8A and B). Furthermore the virulence of Δ*hap2* and Δ*hap5* strains were attenuated in a silkworm infection model (Figure 8C). In the present study, we could not elucidate the function of Hap1p for lactate assimilation. Because *C. glabrata* has two Hap1p, CAGL0K05841g (this study) and CAGL0B03421g, this redundancy might account for the phenotype of Δ*hap1* single deletion in KUE16015. Collectively, we conclude that at least Hap2p and Hap5p transcription factor regulate lactate assimilation and virulence.

In the present study, hyperglycemic silkworms [16] and mice were used for the virulence study because the hyperglycemia is known to be a clinical risk factor of *C. glabrata* infection [32], [33] and *C. glabrata* vaginitis can be detectable in hyperglycemic mice rather than healthy mice [34]. Indeed, hyperglycemic silkworms are killed quicker than healthy silkworm by *C. glabrata* infection and hyperglycemic mice have higher fungal burden than healthy mice following *C. glabrata* oral inoculation (Ueno et al., manuscript in preparation). Even though the hyperglycemic model was used, high dose infection (10^7^∼10^8^ CFU/larvae) was required to analyze mortality due to a low virulence of *C. glabrata*. In general, high dose infection can cause physical damage to the host, and this may confound the virulence. However comparable infection dose of *S. cerevisiae* (10^8^ CFU/larvae) or heat-killed *C. glabrata* dose not cause a mortality in the silkworm infection model (Ueno et al., manuscript in preparation). This suggests that the infection dose of *C. glabrata* was appropriate and LD_50_ from a silkworm infection model reflects the *C. glabrata* virulence rather than direct physical damage.

Carbon availability may be influenced by the infection model employed. The glucose dimer trehalose is a major sugar in insect hemolymph, but 10 mg/mL (1%) of glucose is detected in hemolymph of hyperglycemic silkworms, but not healthy silkworms [16]. Because the glucose leve is gradually decreased by homeostasis in silkworms [16], this glucose concentration may be easily exhausted. Indeed, Min medium containing more than 2.5% glucose stably supports *C. glabrata* growth, but not less than 1.0% glucose (data not shown). If *C. glabrata* infects hyperglycemic silkworms, *C. glabrata* may require lactate assimilation under this condition. The virulence attenuation of the Δ*cyb2* strain is also observed in a healthy silkworm as well as a hyperglycemic silkworm (data not shown). To evaluate gastric colonization of yeast in mice, we used the purified diet AIN-93G and antibiotic doxycycline as described previously [25], [35]. This condition provides a decreased number of intestinal bacteria and reduced amount of organic acids including lactate. As a result, the decreased organic acids can not fully exercise anti-microbial function, and allows *C. albicans* to survive in the intestine [25]. Importantly, treatments including AIN-93G or antibiotics does not completely remove intestinal flora, and organic acids remain in the intestine [25], [35]. Organic acids have a dual nature acting as both an alternative carbon source and exerting anti-microbial activity. Thus it is conceivable that *C. glabrata* could survive by assimilating residue organic acids such as lactate under decreased stress of organic acids in this experiment.

Further study is required to define whether lactate assimilation is important for systemic infection as well as intestinal infection. The *cyb2* mutant was identified as having attenuated virulence in systemic infection of silkworms (Figure 2). This result encourages our speculation that Δ*cyb2* mutant may attenuate virulence in systemic infection of mice. Our speculation also may be supported by the findings that the transcription of *C. albicans CYB2* and lactate transporter *JEN1* were highly upregulated in mouse macrophage[10]. In summary, we describe a new aspect that the commensal pathogen *C. glabrata* requires lactate assimilation for adaptation in the intestine. Further research is required because few factors are known that are required for *Candida* to survive in an intestine.

## Methods

### Media

*C. glabrata* strains were routinely grown at 37°C on YPD [1% (wt/vol) yeast extract, 2% (wt/vol) bacto peptone, 2% (wt/vol) dextrose] or Min [0.17% (wt/vol) yeast nitrogen base without amino acids and ammonium sulfate, 0.5% (wt/vol) ammonium sulfate, 2% (wt/vol) dextrose] media. For solid media, 1.5% (wt/vol) agar was added, unless otherwise noted. Other growth supplements were added as needed to the Min medium per standard protocol [36]. YRM (yeast recovery medium) contained 1.5% (wt/vol) agar, 0.5% (wt/vol) yeast extract, 1% (wt/vol) polypeptone, 1% (wt/vol) dextrose, 100 units/ml penicillin and 100 µg/ml streptomycin sulfate. Lactate, acetate and pyruvate medium contained 0.17% (wt/vol) yeast nitrogen base without amino acids and ammonium sulfate, 0.5% (wt/vol) ammonium sulfate and 2% (vol/vol) L-Lactate for lactate medium, 4% (wt/vol) sodium acetate trihydrate for acetate medium, or 2% (vol/vol) pyruvate as a sole carbon source. Skim milk plates [0.17% (wt/vol) yeast nitrogen base without amino acids and ammonium sulfate, 0.5% (wt/vol) ammonium sulfate, 2% (wt/vol) dextrose, 3% (wt/vol) skim milk, 1.5% (wt/vol) agar] were used to assay the secretion of protease [37]. The egg-yolk plates [1% (wt/vol) peptone, 4% (wt/vol) dextrose, 6% (wt/vol) NaCl, 0.0056% (wt/vol) CaCl_2_, 10% (vol/vol) egg-yolk emulsion (Merck), 1.5% (wt/vol) agar] were prepared as described [38]. *CYB2*-induction medium contained 1% (wt/vol) Yeast extract, 1% (wt/vol) Bacto peptone, 0.1% (wt/vol) dextrose, 2% (vol/vol) D, L-lactate and 2% (vol/vol) ethanol [29].

### Sequence analysis

The amino acid or nucleic acid sequences were obtained from UniprotKB (http://www.uniprot.org/uniprot/), *Saccharomyces cerevisiae* Genome Database (SGD; http://www.yeastgenome.org/) and Genolevures (http://cbi.labri.fr/Genolevures/) [39]. Multiple sequence alignment was performed using ClustalW with default settings at GenomeNet Server (http://align.genome.jp/). BOXSHADE 3.21 server (http://www.ch.embnet.org/software/BOX_form.html) was used to print and shade the multiple alignments. To identify the binding site of transcriptional factor, web tool MATCH™ (http://www.gene-regulation.com/) was used.

### Plasmid construction

The plasmids used in this study are listed in Table S2. To construct pZeoi_comp606, the non-coding region on chromosome F position 605,901 - 606,015 was amplified using the primers pChrF606_F2_EcoRI and pChrF606_R2_BamHI and using wild type genomic DNA as a template. PCR products and the parent plasmid pTEF1/ZEO were digested with the restriction enzymes EcoRI and BamHI. Both fragments were ligated with T4 DNA ligase to construct pZeoi_comp606. For pZeoi_CgCyb2, CAGL0K10736g ORF with its 500-bp promoter and 200-bp terminator was amplified using the primers p11538compF1_BamHI and p11538compR2_BamHI and using wild type genomic DNA as a template. Amplified products and the parent vector pZeoi_comp606 were digested with BamHI. The digested vector was dephosphorylated to protect against self-ligation, and then the insert and vector were ligated to construct pZeoi_CgCyb2. The sequences of primer used in this study are provided in Table S1.

### Strain construction

The strains used in this study are shown in Table 1. The target gene was replaced by the *CgHIS3* marker in following five mutants; KUE11538 (Δ*Cgcyb2,* Acs#; CAGL0K10736g), KUE13139 (Δ*Cgadh1,* Acs#; CAGL0I07843g), KUE16015 (Δ*Cghap1*, Acs#; CAGL0K05841g), KUE13633 (Δ*Cghap2*, Acs#; CAGL0H07843g) and KUE14440 (Δ*Cghap5*, Acs#; CAGL0K09900g). The replacement cassette was prepared by PCR using following primers; p11538F and p11538KR for KUE11538, p13139F and p13139KR for KUE13139, p16015F and p16015KR for KUE16015, p13633F and p13633KR for KUE13633, and p14440F and p14440KR for KUE14440. The plasmid pHIS906 was used as a template. Transformations were preformed by the previous described method [40]. To confirm the intended recombination in transformants, PCR was carried out using forward primer pTET12F and following reverse primers were used; p11538kcheck for KUE11538, p13139kcheck for KUE13139, p16015kcheck for KUE16015, p13633kcheck for KUE13633, and p14440kcheck for KUE14440.

**Table 1:** Strains.

Strain	Parent	Description	Reference
CBS138	-	*C. glabrata* laboratory standard strain, for genome project	[39]
S288C	-	*S. cerevisiae* laboratory standard strain, for genome project	[47]
SC5314	-	*C. albicans* laboratory standard strain, for genome project	[48]
KUE100	2001H	Parent strain, histidine auxotroph, the recipient enable high efficient gene targeting which *yku80* is repressed with *SAT1* flipper	[40]
KUE11538	KUE100	Δ*Cgcyb2* strain, *CgCYB2* (Acs# CAGL0K10736g) was replaced with *CgHIS3* marker	This study
KUE11538BV	KUE11538	Δ*Cgcyb2* with the empty vector pZeoi_comp606, integrated at a non-coding locus of chromosome F, position 605,901 - 606,015	This study
KUE11538CV	KUE11538	Δ*Cgcyb2* with the complement vector pZeoi_CgCyb2, integrated at a non-coding locus of chromosome F, position 605,901 - 606,015	This study
KUE13139	KUE100	Δ*Cgadh1* strain, *CgADH1* (Acs# CAGL0I07843g) was replaced with *CgHIS3* marker	This study
KUE16015	KUE100	Δ*Cghap1* strain, *CgHAP1* (Acs# CAGL0K05841g) was replaced with *CgHIS3* marker	This study
KUE13633	KUE100	Δ*Cghap2* strain, *CgHAP2* (Acs# CAGL0H07843g) was replaced with *CgHIS3* marker	This study
KUE14440	KUE100	Δ*Cghap5* strain, *CgHAP5* (Acs# CAGL0K09900g) was replaced with *CgHIS3* marker	This study
KUE100_chr464	KUE100	Control strain, *CgHIS3* marker was ectopically integrated at a non-coding locus of chromosome I, position 464,986 - 465,003	This study
KUE100_chr606	KUE100	Control strain, *CgHIS3* marker was ectopically integrated at a non-coding locus of chromosome F, position 605,901 - 606,015	This study

Two control strains KUE100_chr464 and KUE100_chr606 were constructed to match the genetic background of the above recombinant strains, which the *CgHIS3* marker integrated into an unrelated locus on chromosome I, position 464,986 - 465,003 and chromosome F, position 605,901 - 606,015 respectively, which is a non-coding region. The integration cassettes were prepared by PCR using pHIS906 as a template, and the primers pchrI464F and pchrI464R for KUE100_chr464, and pchrF606F and pchrF606R for KUE100_chr606. The *CgHIS3* integrations were confirmed by PCR, which primers pTET12F and pchrI464check were used for KUE100_chr464, and pTET12F and pchrF606check for KUE100_chr606.

KUE11538BV and KUE11538CV have the blank vector pZeoi_comp606 and the complement vector pZeoi_CgCyb2, respectively. To transform KUE11538, each vector was amplified using the primers pChr606 F1 and pChr606 R1, and the cassette was integrated into the non-coding region on chromosome F position 605,901 - 606,015. PCR was carried out to confirm intended recombination using the primers pZeoORFcheckF1 and pchr606check, and to confirm the perfect deletion of CAGL0K10736g ORF using the primers p11538qF(1384) and p11538qR(1580). KOD-plus- polymerase (TOYOBO) and Go Taq® Green Master mix (Promega) were used for the cassette PCR and the confirmation PCR for transformants respectively in accordance with manufacturers protocols. The sequences of primer used in this experiment are given in Table S1.

### Infection study

We used a hyperglycemic host to observe *Candida* infection because the hyperglycemia is known to be clinical risk factor of *C. glabrata* infection [32], [33] and *C. glabrata* vaginitis can be detectable in hyperglycemic mice rather than healthy mice [34]. The silkworm infection study was performed as described previously [13], [14], and we followed the protocol of infection study using hyperglycemic silkworms (Matsumoto *et al.*, in preparation). To induce hyperglycemia, silkworms (Hu • Yo × Tukuba • Ne; purchased from Ehime sansyu) were induced by the feed SILKMATE 2S (Nosan Corporation, Yokohama, Japan) including glucose at the first day of fifth-instar larvae [16]. Cells from an overnight culture in YPD medium were harvested and resuspended in saline. On the second day of fifith-instar larvae, 50 µl of suspension was infected into the hemolymph through the dorsal surface of the larvae using a 27-gauge needle. After injection, infected larvae were kept in 37°C.Silkworms were not fed after the injections. LD_50_, 50% lethal dose was determined at 24 h after injection.

Gastric colonization in mice was observed as described previously [25], [35]. The drinking water included 2 mg/ml doxycycline (DOX) and 5% (wt/vol) glucose and purified diet AIN-93G (CLEA Japan) were used to decrease the gastrointestinal bacterial flora and to facilitate gastric colonization of yeast. Male mice (8 weeks old, C57BL/6JJcl; purchased from CLEA Japan) were used. To induce hyperglycemia, 210 mg/kg streptozotocin (STZ; purchased from Wako) was intraperitoneally administered, which injures specifically pancreatic Langerhans cells [41], [42]. Ten mice was used to induce hyperglycemia and five mice with 250∼500 mg/dL blood glucose are selected for the infection after seven days STZ administration. The yeast suspension 250 µL in saline was intragastrically inoculated using a metallic gastric tube. Then 13 days after the inoculation, three mice having averaged body weight in the group were euthanized and killed by cervical dislocation. The three ceca were extirpated without washing inner feces, homogenized together, diluted with saline, and spread onto the YRM plates to determine the number of CFU of each strain in ceca. The experiment was carried out independently two times.

The animal experiments complied with all relevant guidelines and policies of the Animal Welfare Committee of the Faculty of Medicine of Chiba University, Japan (approval ID; DO22-3 [mouse] and DO22-4 [silkworm]).

### Growth analysis and assimilation test

To observe the growth of *C. glabrata,* solid media and liquid media were used. For growth test using solid media, the standard streak method was adopted. AnaeroPack®-anaerobic5% (Mitsubishi Gas Chemical CO. Inc., Japan) was used for anaerobic cultivation. The proliferation in liquid medium was monitored using Biophotrecorder TVS062CA (ADVANTEC), and then the doubling time was calculated by the standard method [36].

Media containing L-lactate, acetate and pyruvate respectively as a sole carbon source were used to test the assimilation ability of strains. For hypoxic cultivations, lactate plates were cultivated in AnaeroPack®-anaerobic5% (Mitsubishi Gas Chemical CO. Inc., Japan) for 6 days at 37°C, and then were cultivated in AnaeroPack®-microaero (Mitsubishi Gas Chemical CO. Inc., Japan) for 3 days at 37°C.

The 96-well cell culture plates were used to test the assimilation ability of strains in liquid medium. It was a triplicate experiment on three occasions. For the dilution series, 100 µL of the lactate, acetate or pyruvate medium were firs added to wells. Strains were cultivated in YPD at 30°C and cells from the overnight culture were harvested and adjusted to 4×10^4^ cells per 100 µL. Then 100 µL of cell suspension was dispensed in each well. The 96-well plates were incubated at 37°C and chronologically observed using an EMax precision Microplate Reader (Molecular Devices). For hypoxic cultivations, AnaeroPack®-microaero was used.

### MIC determination

The sensitivity to nitric oxide (DETA NONOate; Cayman Chemical) and L-lactate were evaluated by the micro-dilution method based on the CLSI (http://www.clsi.org/) standard [43]. In this assay, 96-well titer plates were used in duplicate on two occasions. For the dilution series, reagent of 100 µl solution per well was firstly prepared in Min or YPD medium. The overnight culture was diluted to 4×10^4^ cells/ml by using the same medium. Then 100 µl of the diluted culture was added to the 96-well (200 µl solution per well at this time and start concentration was 4×10^3^ cells per well). The plate was incubated for 24 h at 37°C (no shaking). After the cultivation, the OD_650_ value of each well was determined by an EMax precision Microplate Reader (Molecular Devices). Then the growth rate of each wells were calculated by following formula: 2-well average OD_650_ value at the well containing each concentration of the reagent/2-well average OD_650_ value at the well without the reagent × 100 (%). The MIC value was determined at the lowest concentration of the bottom line of the growth curve.

### Protease, lipase and adherence assay

To assay the secretion of protease and lipase, *C. glabrata* strains were inoculated by streak on to skim milk plates and egg-yolk plates, respectively. These plates were incubated for 2 days at 37°C. The size of the halo around colonies was measured.

The adherence assay was carried out as described previously [44]. Briefly, Cells from an overnight cultures in the *CYB2*-induction medium were harvested and adjusted to OD_600_ of 10∼20. Then 200 µl of suspensions were dispensed into a polystyrene 96-well plate (Nunclon™ Δ #163320; round bottom). The plate was incubated for 24 h at 37°C. Non-adherent cells were removed by washing the plate gently two times with distilled water. Then 100 µl of saline and 50 µl of activated XTT solution (Biological Industries) were serially added and the plate was incubated for 6 h at 37°C. A colorimetric change was measured at 490 nm using an EMax precision Microplate Reader (Molecular Devices).

### Quantitative real time reverse transcription-PCR (qRT-PCR)

Four strains, the wild type, *hap1*, Δ*hap2* and Δ*hap5* strains, were cultivated for overnight in 15 mL of YPD media at 37°C. Cells from overnight cultures were harvested and washed in saline two times. Before the induction, 5 mL out of 15 mL suspension was harvested as the first sample, and 10 mL of suspension was harvested and resuspended in 40 mL of the *CYB2*-induction medium to induce *CYB2* transcription. Then, 40 mL suspension was dispensed to four 50-mL polypropylene tubes and shaken at 37°C to start the induction. After induction, Cells from 1 and 3 hr cultures were also harvested. Total RNA from all cultivated samples were prepared using RNA-bee™ (Tel Test Inc.). To quantify mRNA, we used TURBO DNA-free™ kit (Ambion) for DNase treatment, PrimeScript RT reagent Kit (TaKaRa) for the synthesis of cDNA and SYBR® Premix Ex Taq™ II for quantitative PCR. The real-time PCRs for *ACT1* and *CYB2* were carried out using primers ACT1 59F and ACT1 249R for *ACT1*, p11538qF(1384) and p11538qR(1580) for *CYB2,* and ABI PRISM 7000 (Applied Biosystems) as a detection system. The sequences of primer used in this experiment are given in Table S1.

## Supporting Information

Figure S1 **Map of gluconeogenesis and related metabolism.** For detail, please see previous reports [6], [7]. This map is from *S. cerevisiae and C. albicans* study. Note that *C. glabrata* does not have lactate a transporter Jen1p orthologue, although CaJen1p was identified in *C. albicans* [11]. Instead, putative transporters have been found in the *C. glabrata* genome which have homology to the pyruvate transporter or human muscular lactate transporter.
(TIFF)

Figure S2 **Functional domain and sequence alignment of Cyb2p.** The upper figure represents the functional domains of ScCyb2p. The numbers are amino acid positions. Amino acid alignment was performed between ScCyb2p and CgCyb2p. Dark grey box highlights and light grey box indicates identical residues and conserved residues respectively.
(TIFF)

Table S1 **Primers.**
(DOC)

Table S2 **Plasmids.**
(DOC)
