# Supplementary figures and images for: Intestinal Resident Yeast Candida glabrata Requires Cyb2p-Mediated Lactate Assimilation to Adapt in Mouse Intestine
Source: PLoS One. 2011 Sep 9;6(9):e24759. doi: 10.1371/journal.pone.0024759 (PMC3170380; doi:10.1371/journal.pone.0024759)

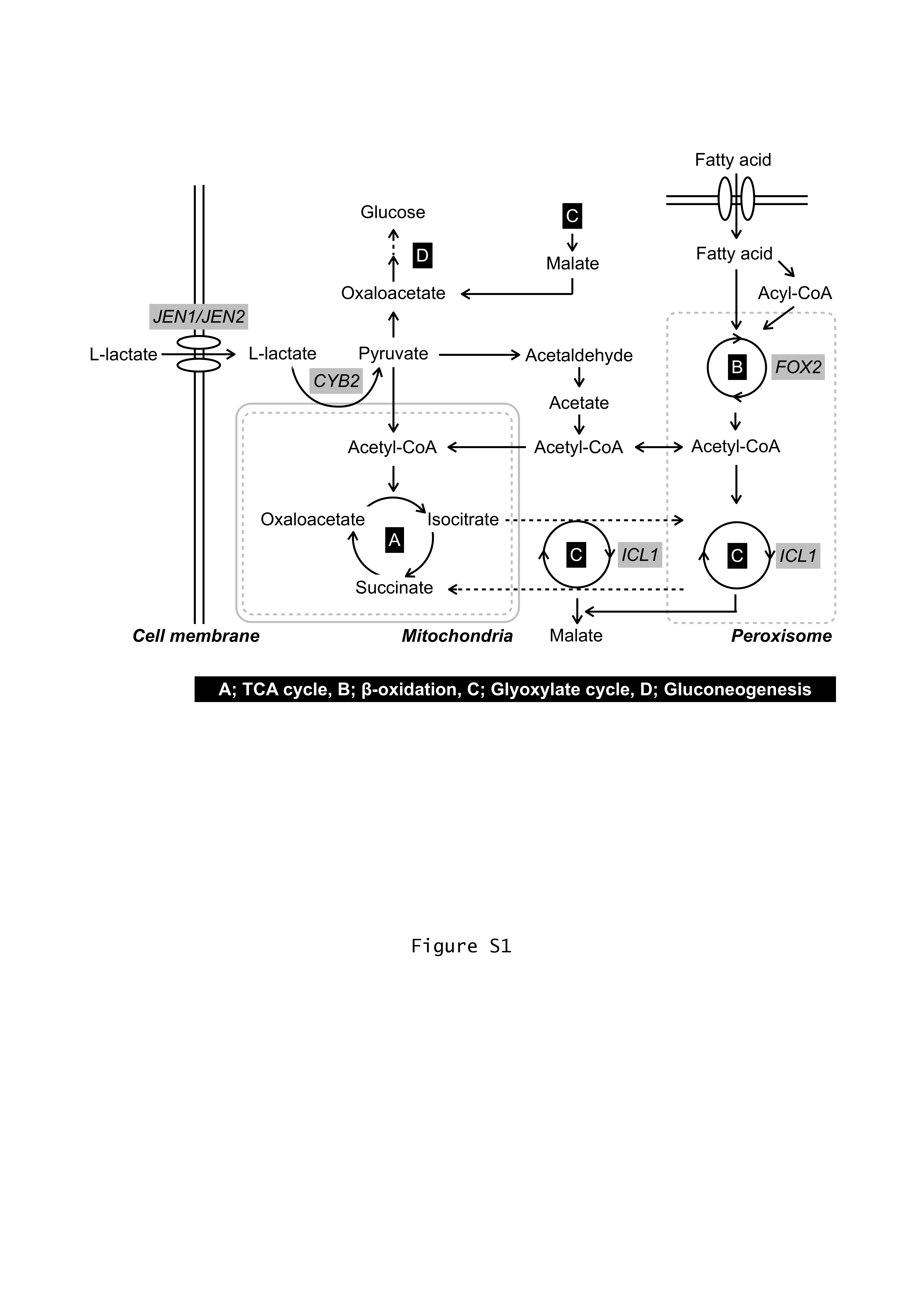

Supplement: Figure S1 — Map of gluconeogenesis and related metabolism. For detail, please see previous reports [6], [7]. This map is from S. cerevisiae and C. albicans study. Note that C. glabrata does not have lactate a transporter Jen1p orthologue, although CaJen1p was identified in C. albicans [11]. Instead, putative transporters have been found in the C. glabrata genome which have homology to the pyruvate transporter or human muscular lactate transporter. (TIFF) [file pone.0024759.s001.tiff]

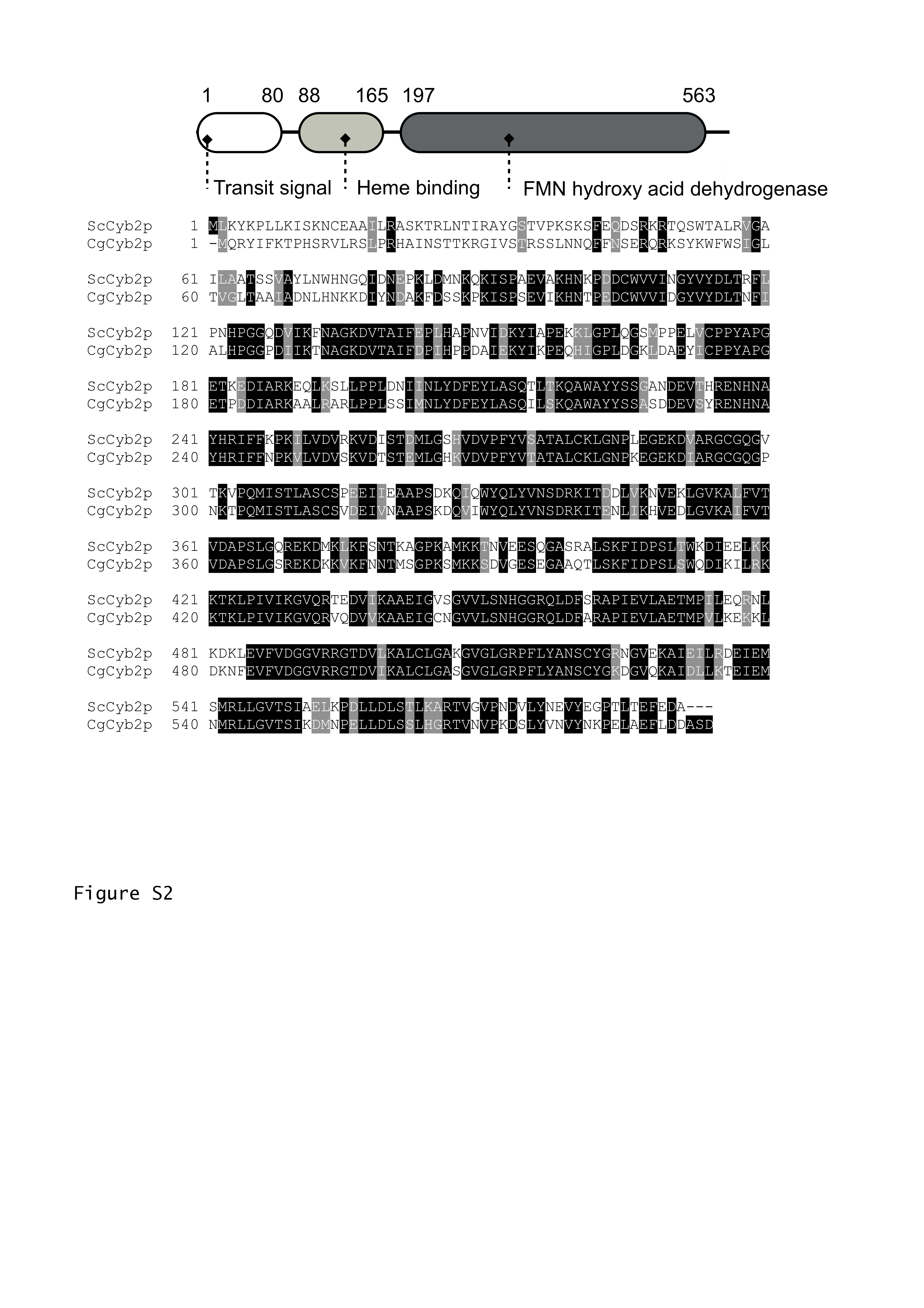

Supplement: Figure S2 — Functional domain and sequence alignment of Cyb2p. The upper figure represents the functional domains of ScCyb2p. The numbers are amino acid positions. Amino acid alignment was performed between ScCyb2p and CgCyb2p. Dark grey box highlights and light grey box indicates identical residues and conserved residues respectively. (TIFF) [file pone.0024759.s002.tiff]
