# Supplementary material for: Intestinal Resident Yeast Candida glabrata Requires Cyb2p-Mediated Lactate Assimilation to Adapt in Mouse Intestine
Source: PLoS One. 2011 Sep 9;6(9):e24759. doi: 10.1371/journal.pone.0024759 (PMC3170380; doi:10.1371/journal.pone.0024759)
Supplement: Table S1 — Primers. (DOC) [file pone.0024759.s003.doc]

### Table S1. Primers

| Name | Sequence | Use |
| --- | --- | --- |
| pChrF606_F2_EcoRI | AGAATTCAGGCTTTAATCAATGTGACGAACTATGCGAAGGAAATTC | Construction of pZeoi_comp606 |
| pChrF606_R2_BamHI | TGGGATCCTTCTGATCCTCTTTTACTGCAACTGTATGGCGCTTG |  |
| p11538compF1_BamHI | ACTGGATCCCCGACGTATAGTTTCCCCTAAGGGC | Construction of pZeoi_CgCyb2 |
| p11538compR2_BamHI | TTGGATCCCGAGAATCACTAACGCAATTGAAGACAGCTTGCAG |  |
| p11538F | TATATTCTGAACTTTATATAGTATAAGGAAAGTATCAAGACAGAAAGAAAAATATA**GGCCGCTGATCACG** | Construction of KUE11538 |
| p11538KR | ATAGTGATACTTTTATACTTACTTAAGAATAAATGAATGTATCGATTTCAAAGTCA**CATCGTGAGGCTGG** |  |
| p11538kcheck | AAGAACGCCATCTACCACGG |  |
| p13139F | TACTTACAGAGAGAGAAACAAGAACATTTTTAAATTCTGCAAAAAACACATAAACA**GGCCGCTGATCACG** | Construction of KUE13139 |
| p13139KR | AGAAAAAACAAATCACTTTCTTTTTTTGGATTTAGAATTTCAAATTAAGCATCTTA**CATCGTGAGGCTGG** |  |
| p13139kcheck | TGCCTTAGGTGCCGTTAGTG |  |
| p16015F | AAAGAGCCATTTTATATTGCAAAGAGAGGAACAATAATCTACGGATCTAAATCCTG**GGCCGCTGATCACG** | Construction of KUE16015 |
| p16015KR | CACAATTAAATATTATATATCCTTTCTAAAGCCTGATTATATTCCACTAAGAATTA**CATCGTGAGGCTGG** |  |
| p16015kcheck | GGTATTGCCTTTGGACTGCC |  |
| p13633F | GTCCAGGAACTATTTACCTTTAAACATCAAGCTCATACAAATACGACGGCATTAAC**GGCCGCTGATCACG** | Construction of KUE13633 |
| p13633KR | TTGAAGTCACTTGCATTACAAAATAATGCTGCTCAAACTTTAGCCCACCTGAGTTA**CATCGTGAGGCTGG** |  |
| p13633kcheck | CTCCACTGAGTTATCTGAGG |  |
| p14440F | TTACAAAGGCAGAGTACTGCACGGTACTGCACAGGTTTATATATTAGAAGTGGGTA**GGCCGCTGATCACG** | Construction of KUE14440 |
| p14440KR | TTGCTTCTCTTGTCCTTGCATGTATGTACTTATTCAGGATTACATACTACATATTA**CATCGTGAGGCTGG** |  |
| p14440kcheck | CATACTGGAATCAGGCACCG |  |
| pTET12F | AGAAAACCAGCCTCACGATG | Confirmation of correct cassette integration in mutants |
| pChr606 F1 | AAGAATGCCAACCAAGGATTCACAATAATCCGAAGC | Construction of KUE11538BV and KUE11538CV |
| pChr606 R1 | TTAGGCAAAGCATTTGTAAACCATTACAAGCACTC |  |
| pZeoORFcheckF1 | AAGTTGACCAGTGCCGTTCCGGTG |  |
| p11538qF(1384) | GCTCCGATTGAAGTTCTTGC | The confirmation of complete deletion for *CgCYB2* ORF |
| p11538qR(1580) | TGCACACCATCCTTTCCATA |  |
| ACT1 59F | ACAACGGTTCCGGTATGTGT | qRT-PCR |
| ACT1 249R | ACCGTGTTCGATTGGGTAAC |  |
| pchrI464F | ACGGGGTACGACAAAACTATCCAAAGAATGAGATTGCCAATCCTTATTTTCTGAATGGCCGCTGATCACG | Construction of KUE100_chr464 |
| pchrI464R | AATTCTTGCTGAATATGAAGCAGTCGTTTCTTCATACAGAACAGTAAGTAAAATAACATCGTGAGGCTGG |  |
| pchrI464check | AACTTCTTGGTTGAAAACCCTACAC |  |
| pchrF606F | AGGCTTTAATCAATGTGACGAACTATGCGAAGGAAATTCTTGTACAAAAATGGACAGGCCGCTGATCACG | Construction of KUE100_chr606 |
| pchrF606R | TTGCTATCCTTACTACTAGAATCTTCACAAATTGGGTATGTGTAACCTGATATCATCATCGTGAGGCTGG |  |
| pchrF606check | CTAATGGGGATATAGAAAGATAGGG |  |
