# Supplementary material for: Intestinal Resident Yeast Candida glabrata Requires Cyb2p-Mediated Lactate Assimilation to Adapt in Mouse Intestine
Source: PLoS One. 2011 Sep 9;6(9):e24759. doi: 10.1371/journal.pone.0024759 (PMC3170380; doi:10.1371/journal.pone.0024759)
Supplement: Table S2 — Plasmids. (DOC) [file pone.0024759.s004.doc]

### Table S2. Plasmids

| Name | Description | Source or reference |
| --- | --- | --- |
| pHIS906 | Carrying *CgHIS3* marker, PCR template for the cassette amplification | Ueno *et al.,* in preparation |
| pTEF1/ZEO | Carrying Zeocin^R^ marker, parent of pZeoi_comp606 | Invitrogen, (Alderton *et al.*, 2006) |
| pZeoi_comp606 | The blank vector for revertant construction, parent of pZeoi_Cgcyb2, used to integrate at a no-coding region on chromosome F, position 605,901 - 606,015 | This study |
| pZeoi_CgCyb2 | Carrying *CgCYB2* ORF and 500 bp of 5’ sequence, used to make revertant | This study |

**Alderton, A. J., Burr, I., Mühlschlegel, F. A. & Tuite, M. F. (2006).** Zeocin resistance as a dominant selective marker for transformation and targeted gene deletions in Candida glabrata. *Mycoses* **49**, 445-451.
